# The effect of the COVID-19 pandemic on orthognathic surgery in Sweden - a registry-based study

**DOI:** 10.1186/s12903-026-07651-w

**Published:** 2026-01-12

**Authors:** Carina Cardemil, Adrian Salinas Fredricson, Lars Rasmusson, Lillemor Dimberg

**Affiliations:** 1 Dep of Oral and Maxillofacial Surgery and Jaw Orthopedics, Karolinska University Hospital, Stockholm, Sweden; 2 Department of Biomaterials, Institute of Clinical Sciences, Sahlgrenska Academy at University of Gothenburg, Gothenburg, Sweden; 3 Department of Oral and Maxillofacial Surgery, Eastmaninstitutet, Folktandvården Stockholm AB, Stockholm, Sweden; 4 Division of Oral Diagnostics and Rehabilitation, Department of Dental Medicine, Karolinska Institutet, Stockholm, Sweden; 5 Department of Oral and Maxillofacial Surgery, The Sahlgrenska Academy, University of Gothenburg, Gothenburg, Sweden; 6 Maxillofacial Unit, Linköping University hospital, Linköping, Sweden; 7 Head and Neck Services, York Teaching Hospital, York, United Kingdom; 8 Swedish Agency for Health Technology Assessment and Assessment of Social Services, Stockholm, Sweden; 9 Health Technology Assessment Odontology (HTA-O), Faculty of Odontology, Malmö University, Malmö, Sweden

**Keywords:** COVID-19, Orthognathic surgery, Epidemiology, Registries

## Abstract

**Background:**

The COVID-19 pandemic had a noticeable impact on healthcare services. The restrictions imposed during the pandemic where extensive, and in Sweden, elective surgery was deprioritized to make room for the most severely affected patients. This study aimed to evaluate the effect of the COVID-19 pandemic on the volume of orthognathic surgery (OGS) in Sweden, and to compare the impact between regional and university hospitals.

**Methods:**

This registry-based study used national data from the National Quality Registry for Orthognathic Surgery (NROK). OGS performed during 2018–2022 and registered in NROK were included. The follow-up time was divided into three time periods: pre-pandemic, pandemic and post-pandemic. Of 21 hospitals performing OGS in Sweden, 18 (11 regional and seven university hospitals) were included in the final analysis. Differences in national monthly means were analyzed using one-way ANOVA and Kruskal-Wallis tests, with post hoc comparisons. Linear regression was used to assess differences in trends between hospital types.

**Results:**

The national monthly mean of OGS was significantly lower during the pandemic compared to both the pre-pandemic period (− 13.7 surgeries, 95% CI: 3.6–23.8; *p* < 0.01) and the post-pandemic period (− 15.6 surgeries, 95% CI: 2.6–28.7; *p* < 0.05). University hospitals experienced a 40.7% reduction in surgeries during the pandemic (*p* < 0.001), whereas the reduction at regional hospitals (− 19.7%) was not statistically significant (*p* = 0.603). No significant differences were found between the pre- and post-pandemic periods, neither in national monthly means nor when comparing hospital types.

**Conclusions:**

A significant decrease in the number of OGS was observed during the pandemic in Sweden. No statistically significant difference was found between the pre- and post-pandemic periods, suggesting a meaningful normalization in the frequency of OGS in Sweden following the pandemic, although not accounting for possible surgical backlog. University hospitals appeared to experience a greater relative decrease in surgical volume during the pandemic compared to regional hospitals. This difference may be attributable to lower baseline volumes at regional hospitals rather than a differential impact of the pandemic itself.

## Background

Malocclusion is a common condition, affecting 70% of children and adolescents. However, only a significantly smaller group of patients have a malocclusion, breathing problems and/or severe aesthetic malformations that requires orthognathic surgery (OGS) in combination with the orthodontic treatment [1, 2]. In a recent study over a 5-year period, the average prevalence rate of OGS in Sweden was 6.3 per 100,000 persons [3]. In Sweden, OGS is performed in all healthcare regions, mostly in university hospitals but to some extent in regional hospitals. There is a small number of patients with craniofacial anomalies and other pathological conditions, and these patients are specifically referred to university hospitals. In Sweden, OGS is integrated into the national healthcare system and is only performed under national health care insurance.

The combined treatment where orthodontic treatment is followed by surgery is a long and complex treatment, which in general last for approximately 2 years in total. Most cases, commence with orthodontic treatment for over a year. The surgeries are performed in general anesthesia with a duration of around 60–180 min. Most surgeries require inpatient care for at least 24 h. Post-surgical treatment with orthodontic appliances typically lasts for 2–6 months.

The COVID-19 pandemic had a great impact on elective surgery worldwide due to restrictions and prioritization of hospital resources. In the first wave of COVID-19, at least 21 million elective surgical procedures were canceled worldwide because of postoperative SARS-CoV-2 infection concerns for the patients and the capacity of the hospitals [4]. Other reasons for pausing OGS during the pandemic have been high concentrations of SARS-CoV-2 virus in the direct area of the airways [5] and an increased risk of contamination of coronavirus to the staff in the operating room due to aerosol exposure [6]. As a result of the restrictions, patients may have experienced complications due to prolonged treatment [7]. During the time when the world was in lockdown and affected by restrictions to combat the pandemic, few OGS were performed in Swedish hospitals, i.e. from March 2020 to February 2022, which is in accordance with international reports [8, 9].

For patients in need of OGS, this delay may have resulted in emotional suffering while waiting for treatment [9, 10]. Another negative consequence is the strained situation in orthodontic clinics since patients have been retained in the clinic for a prolonged time, with an increased cost for the clinics. According to a Finnish study 30.2% patients in orthodontic care reported unfavorable changes and 9.5% relapsed back to a previous stage in the treatment because of the lockdown during the pandemic [11].

In 2017, the National Registry for Orthognathic Surgery (NROK) was established, enabling longitudinal studies of variations in OGS across Sweden [12]. In the present study, data from NROK was used to describe the effects of the COVID-19 pandemic on orthognathic care during the pandemic years 2020 and 2021. Specifically, the aim was to evaluate the impact of the pandemic on the national volume of OGS and to assess differences between regional and university hospitals. To our knowledge, this is one of the first national registry–based studies to examine OGS activity during the COVID-19 pandemic, providing a comprehensive overview of its impact across both university and regional hospitals in Sweden.

## Methods

This registry-based longitudinal study used data from NROK to collect the number of OGS during the years 2018–2022, to investigate the impact of the COVID-19 pandemic on the number of OGS performed and to assess differences in impact between university and regional hospitals.

### Description of the NROK registry

NROK was initiated in 2017, and the overall purpose of the registry is to ensure the quality of care and to continuously document and evaluate patient benefit and patient safety. Furthermore, the register serves to ensure equal care and form the basis to achieve consensus regarding treatment indications, in addition to contribute to the continued development of OGS care. The registry is accessible to all stakeholders; surgeons, patients, decision-makers, and other health care principals. In Sweden, 21 hospital units perform OGS, including seven university hospitals. When the registry was established, all units performing OGS were connected to NROK. In 2019, one regional hospital discontinued its participation. At the time of the study, 20 hospitals were connected to the registry, including all seven university hospitals. Reporting adherence is reported to be high, with coverage exceeding 90% [12].

### Registry inquiry

All OGS on Swedish residents aged above 18 and registered in NROK during 2018–2022 were included in the study. Data from all 21 hospitals were collected. However, the one regional hospital that had discontinued its participation in NROK, along with two additional regional hospitals with no registrations during the follow-up period, were excluded from the analysis. Surgical events from the remaining 18 hospitals (11 regional and 7 university hospitals) formed the basis of the final dataset. Surgical events with records that did not include information on the type of surgery performed were also excluded. The follow-up period was divided into three different time periods: pre-pandemic (24 months), pandemic (22 months), and post-pandemic (10 months). The pandemic was considered to have begun in March 2020, when the Public Health Agency of Sweden (PHAS) installed the first restrictions on public health care. The post-pandemic period was considered to begin in March 2022 when PHAS had lifted most of the restrictions on the public and health care system.

### Categorization of variables

Skeletal and orthodontic diagnoses were categorized as being either maxillary or mandibular. Maxillary anomalies were considered as maxillary retrognathia, maxillary prognathy, clef lip and palate, maxillary vertical hyperplasia and hypoplasia. Mandibular anomalies were considered as mandibular retrognathia, mandibular prognathy and mandibular asymmetry.

The type of surgery was categorized as either maxillary, mandibular or bimaxillary. Maxillary surgery was comprised of surgically assisted rapid maxillary expansion (SARME), Le Fort I surgery, sectioned Le Fort surgery, Le Fort II surgery, Le Fort III surgery and maxillary osteodistraction. Mandibular surgery was comprised of intraoral vertical ramus osteotomies (IVRO), extraoral vertical ramus osteotomies (EVRO), bilateral sagittal split osteotomy (BSSO), mandibular segmented osteotomies, mandibular distraction osteotomies and genioplasty surgeries. Surgical treatments that included both mandibular and maxillary surgeries performed at the same time were considered as bimaxillary surgeries. The analysis of monthly means included total number of surgeries, regardless of type (maxillary, mandibular, or bimaxillary). To investigate the impact of the COVID-19 pandemic on different type of care providers, surgical units were divided into university hospitals and regional hospitals.

### Statistics

All statistical analyses were performed using STATA/SE 16.1, StataCorp LLC, Texas. As the duration of follow-up differed between the three time periods, monthly means for each time period were used for all comparative analyses. National monthly means of OGS during the pre-pandemic, pandemic and post-pandemic periods were analyzed. Due to minor deviations from normality (Shapiro–Wilk *p* < 0.05) but approximately normal residual distributions on Q–Q plots, both ANOVA and Kruskal–Wallis tests were applied to assess robustness. Post-hoc analyses were performed for pairwise comparisons using Tukey’s HSD and Mann-Whitney U. Linear regression was used to compare the difference in the impact of the pandemic on regional and university hospitals. P-values < 0.05 were considered statistically significant.

## Results

### Study sample

**Fig. 1:**
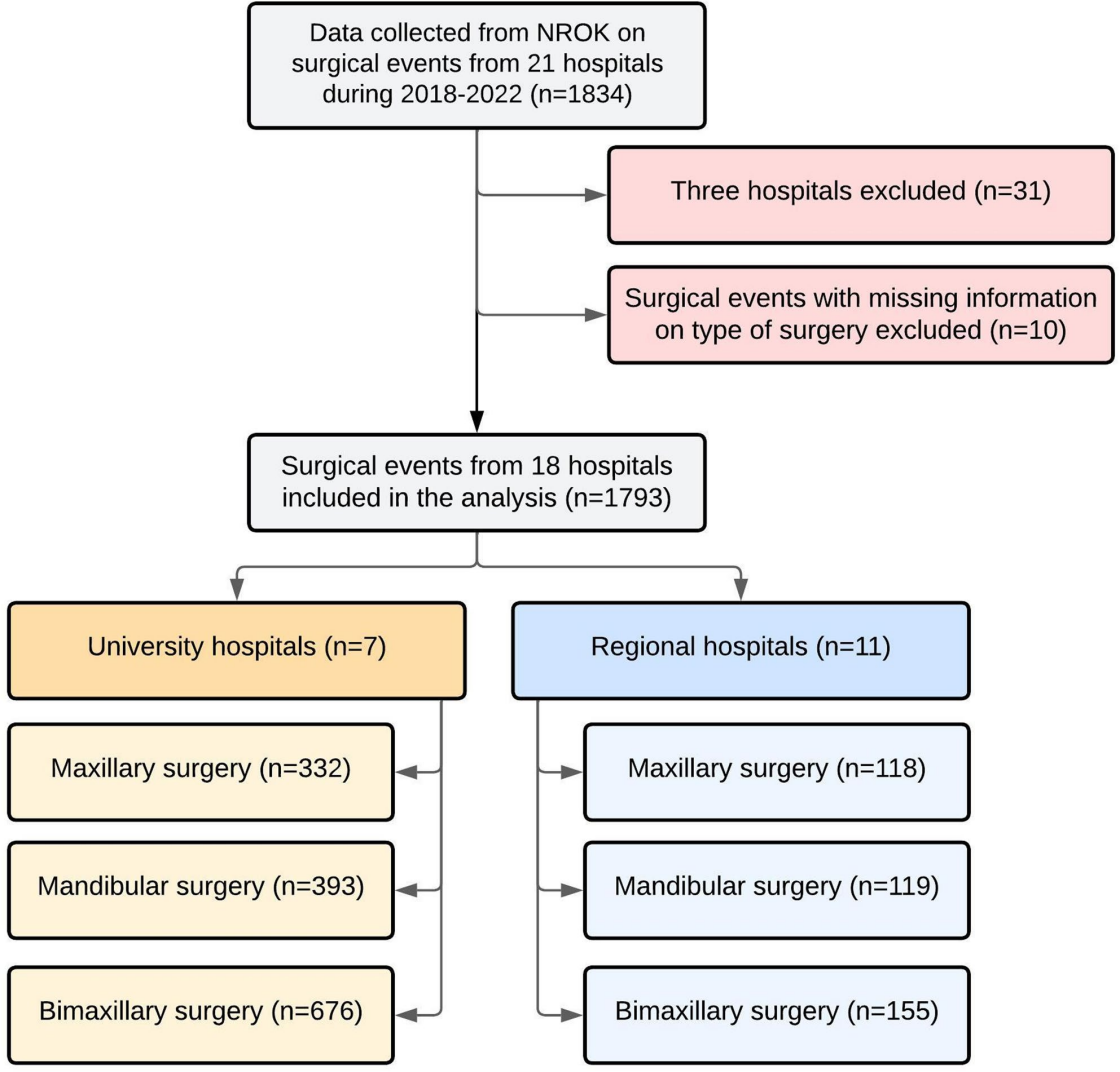
Flowchart presenting the recruitment process, exclusion of hospitals and surgical events as well as distribution of surgeries in regional and university hospitals

**Fig. 2:**
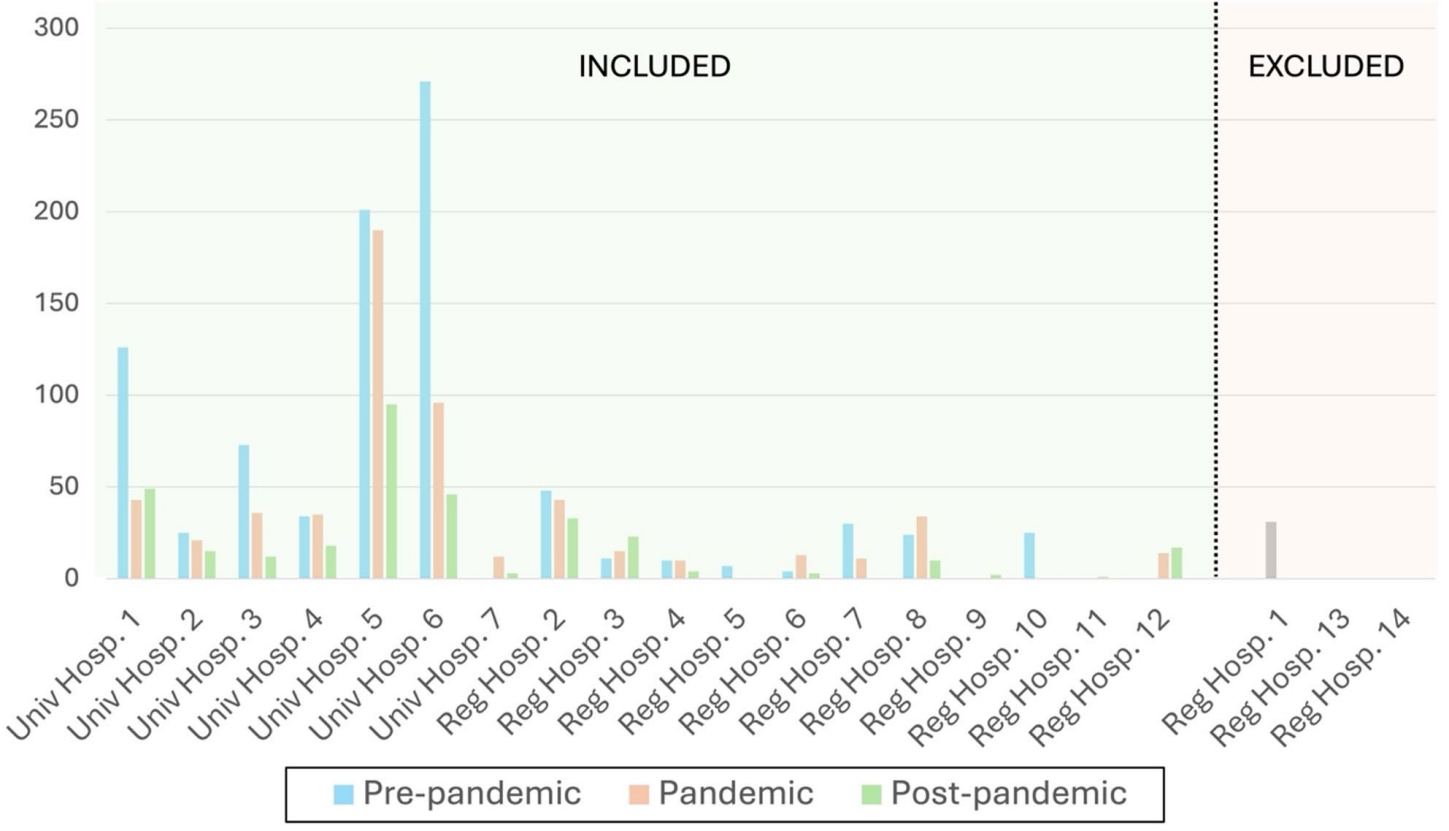
Included and excluded university and regional hospitals, with total number of surgeries during the three time periods

The registry holder excluded all individuals aged under 18, and delivered a data set containing 1834 surgical events. After excluding three hospitals (*n* = 31) and registrations with missing information on type of surgery performed (*n* = 10), the final study sample consisted of 1793 surgeries from 18 hospitals: 11 regional hospitals and seven university hospitals. Among the 1793 surgeries included in the study, 78 (4.5%) were reoperations: 37 maxillary procedures, 14 mandibular procedures, and 27 bimaxillary procedures. Reoperations were included in the analysis of total volume of OGS. The recruitment process is presented in Fig. 1. The properties of the study sample, categorization of diagnostics and types of surgery during each year are presented in Table 1. There was no statistically significant difference in the baseline characteristics. The volume of total surgical events among both included and excluded hospitals is shown in Fig. 2.

**Table 1:** Baseline properties of the study population, types and numbers of surgeries during the time periods and distribution between regional and university hospitals

	Pre-pandemic		Pandemic		Post-pandemic		Total	
	Univ Hosp	Reg Hosp	Univ Hosp	Reg Hosp	Univ Hosp	Reg Hosp	Univ Hosp	Reg Hosp
Age								
Mean	24.5	25.2	25.4	25.2	24.9	25.1	24.8	25.2
IQR 25	19.9	19.8	20.5	20.3	20.9	21.0	20.2	20.4
Median	21.4	21.6	22.6	22.1	22.6	22.3	22.0	22.0
IQR 75	25.7	25.9	27.4	25.2	26.2	25.5	26.3	25.4
Range	18.0–71.9	18.0–69.7	18.1–58.2	18.0–63.6	18.2–52.8	18.3–61.5	18.0–71.9	18.0-69.7
Medical history								
Completely healthy	613	130	334	96	235	91	1182	317
Recorded condition	108	29	63	20	36	25	207	74
Missing	9	0	0	1	3	0	12	1
Diagnostics								
Maxillary discrepancy	370	70	193	55	137	50	700	175
Mandibular discrepancy	505	113	272	77	168	88	945	278
Open bite	170	42	72	28	50	24	292	94
Hemifacial microsomia	5	0	2	0	0	0	7	0
Craniofacial malformation	10	0	2	0	3	0	15	0
Other FMD	41	11	13	5	18	5	72	21
Surgeries								
Maxillary surgery	189	49	82	41	61	28	332	118
Mandibular surgery	207	45	114	33	72	41	393	119
Bimaxillary surgery	334	65	201	43	141	47	676	155
Total operations	730	159	397	117	274	116	1401	392
Reoperations	37	8	14	2	7	10	58	20

*FMD  Facial Morphology Discrepancy

### Impact of COVID-19 on national monthly means

**Fig. 3:**
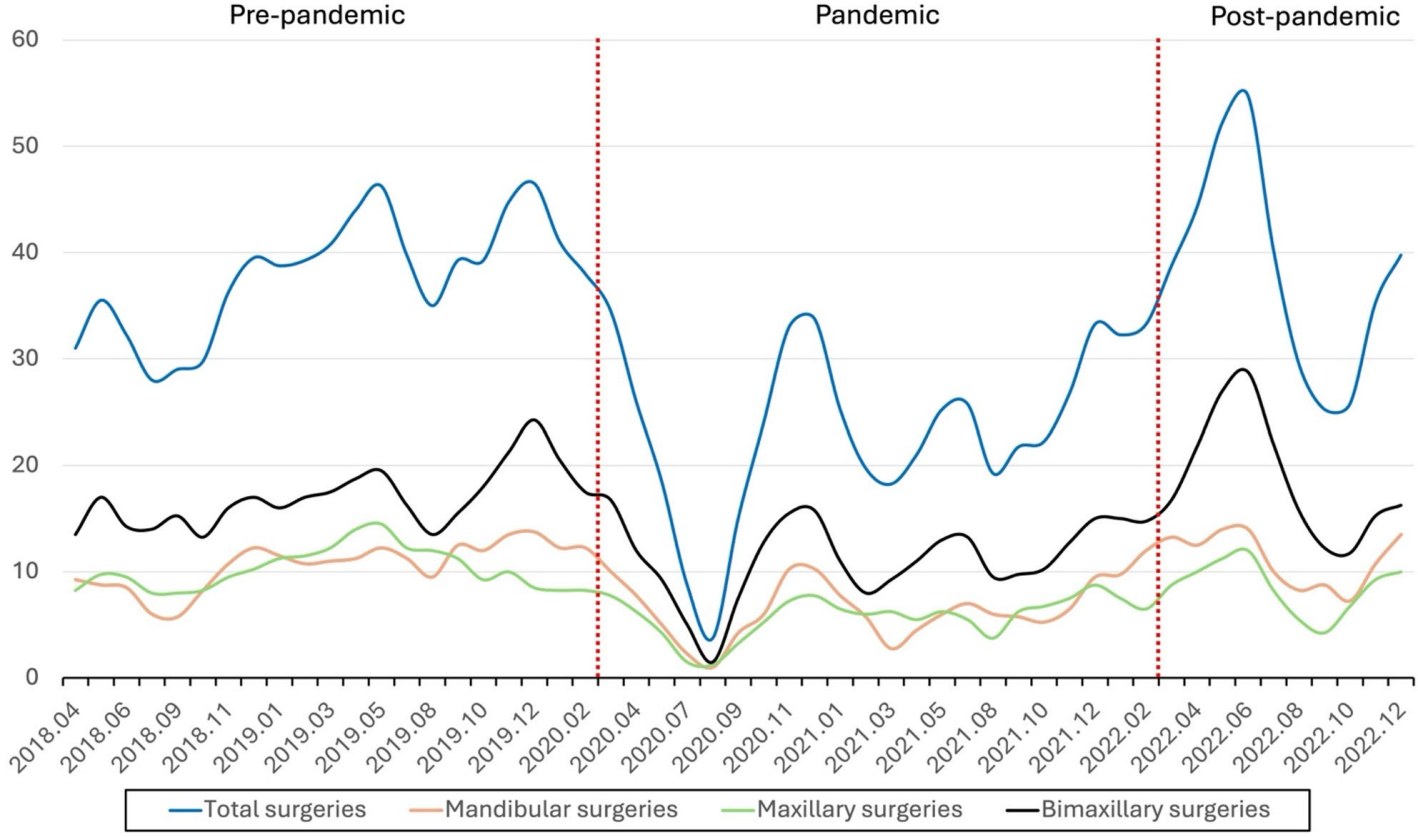
Three-month moving average of monthly surgical volumes during 2018–2022. The pre-pandemic, pandemic, and post-pandemic periods comprised 24, 22, and 10 months, respectively

**Fig. 4:**
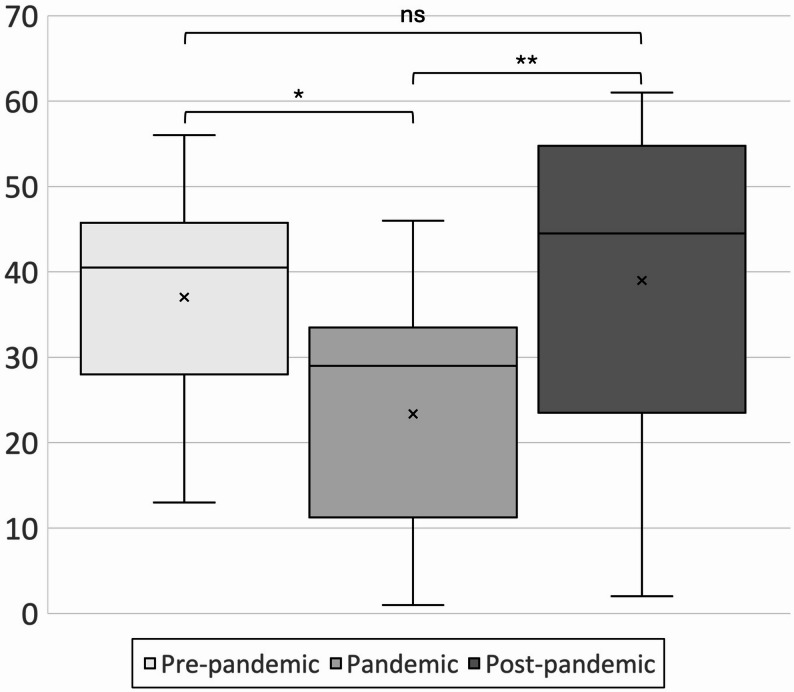
National monthly means of OGS during the three time periods and results from ANOVA and Kruskal Wallis analysis, as well as post hoc Tukey’s HSD and Mann-Whitney U. *= p<0.01, **= p<0.05, ns = non-significant

There was a distinct drop in the number of monthly surgeries shortly after the introduction of the PHAS regulations related to the COVID-19 pandemic. The drop is most clearly visualized in direct conjunction with the newly introduced restrictions in March 2020, to steadily increase over time thereafter, to finally in March 2022 reach monthly levels that are comparable to pre-pandemic data. The national monthly means during the time periods and the differences in means are shown in Table 2. Figure 3 displays the three-month moving average of monthly surgical volumes during 2018–2022.The one-way ANOVA showed a significant effect of the pandemic on the monthly number of surgeries (F = 6.84, *p* = 0.0023). The Kruskal–Wallis test also indicated a significant difference between periods (H = 11.73, *p* = 0.0028) with a moderate effect size (η²≈0.20). Post-hoc analyses using Tukey’s HSD and the Mann–Whitney U test showed significant differences between the pre-pandemic and pandemic periods (*p* = 0.005 and *p* = 0.0015, respectively), and between the pandemic and post-pandemic periods (*p* = 0.015 and *p* = 0.0193, respectively). No statistically significant difference was found between the pre-pandemic and post-pandemic periods. The results from these analyses are presented in Table 2 and visualized in Fig. 4.

**Table 2:** Monthly means of orthognathic surgery during the different time periods, and the differences in means. Pairwise comparisons were conducted with tukey’s HSD and Mann-Whitney U test

Comparison	Mean	SD	Count	Mean difference	SE	95% CI	*p* (T*)	*p* (M-W*)
Pre-pandemic	37.0	11.7	24					
Pandemic	23.4	13.6	22					
Post-pandemic	39.0	19.9	10					
Pre-pandemic vs. Pandemic				13.7	4.2	3.6 23.8	0.005	0.0015
Post-pandemic vs. Pandemic				15.6	5.4	2.6 28.7	0.015	0.0193
Pre-pandemic vs. Post-pandemic				-1.9	5.3	-14.8 10.9	0.928^NS^	0.384^NS^

ns: non-significant

T* = Tukey’s HSD

M-W = Mann-Whitney

### Impact of COVID-19 on regional and university hospitals

**Fig. 5:**
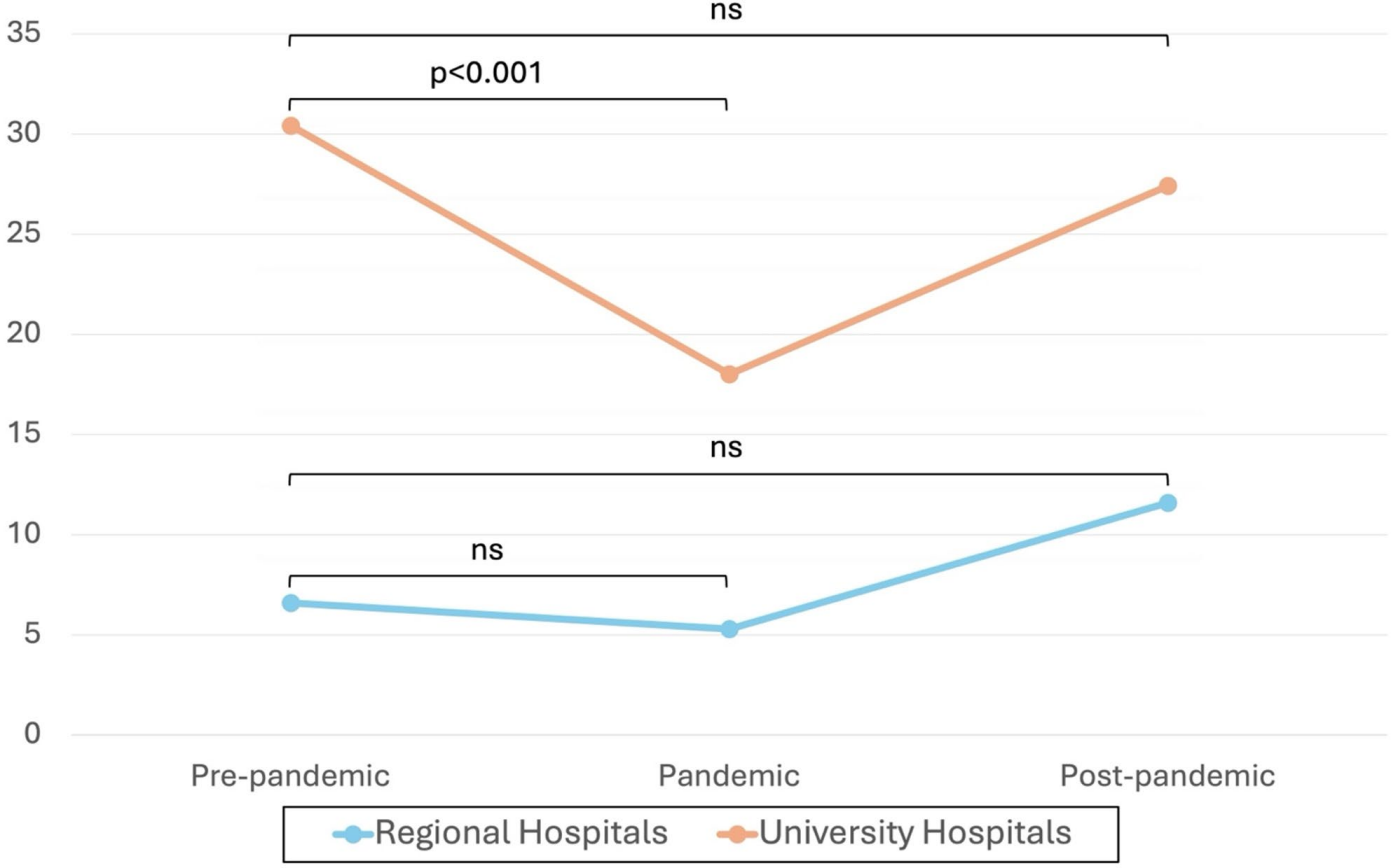
Monthly means of surgeries and changes over time during the time periods, divided on regional and university hospitals. Results from linear regression. ns = non-significant

The linear regression analysis demonstrated differences in how regional and university hospitals were affected during the pandemic. University hospitals experienced a 40.7% reduction in mean monthly surgeries during the pandemic compared to the pre-pandemic period (*p* < 0.001), with no significant differences between pre- and post-pandemic periods (-9.9%, *p* = 0.347). Regional hospitals had a 19.7% reduction in monthly surgeries during the pandemic compared to before, which was not statistically significant (*p* = 0.603). In the post-pandemic period, regional hospitals showed a non-significant increase of 74.9% compared to pre-pandemic levels (*p* = 0.122). The monthly means and changes during the periods are displayed in Table 3 and visualized in Fig. 5.

**Table 3:** Monthly means of orthognathic surgeries in regional and university hospitals respectively, as well as the percentual change during the pandemic. Analyzed using linear regression. All regional (*n* = 11) and university (*n* = 7) hospitals contributed data across all three study periods

	Regional hospitals	95% CI	*p*	University hospitals	95% CI	*p*
Pre-pandemic means	6.6	3.2 10.1		30.4	27.0 33.9	
Pandemic means	5.3	1.7 8.9		18.0	14.5 21.6	
Post-pandemic means	11.6	6.3 16.9		27.4	22.1 32.7	
Δ Pre-pandemic ◊ Pandemic	-1.3 (-19.7%)	-6.3 3.6	0.603	-12.4 (-40.7%)	-17.3 -7.4	< 0.001
Δ Pre-pandemic ◊ Post-pandemic	+ 5.0 (74.9%)	-1.4 11.3	0.122	-3.0 (-9.9%)	-9.4 3.3	0.347

## Discussion

The purpose of this registry-based longitudinal study was to assess how the COVID-19 pandemic affected OGS in Sweden. This study shows how the outbreak of COVID-19 had a direct effect on the hospitals in Sweden, resulting in a sharp decrease in the number of performed OGS, thus confirming a considerable impact of the pandemic on elective surgery. The reduction in elective procedures during the pandemic is consistent with previous studies, which have reported an increased use of outpatient surgery during this period [13, 14]. Not only planned surgeries were reduced, reports on emergency general surgery operations during the time of governmental restrictions have shown a reduction by more than half of the pre-pandemic numbers [15].

In a systematic review of oral and maxillofacial surgery practice during the COVID-19 pandemic, urgent cases and cases of emergency were shown to have been maintained and least affected of the changes in surgical practice [16]. Bartella et al. [17] showed a 45% decrease of the number of surgeries at their maxillofacial department, with no elective surgeries performed during a few weeks at the time of the COVID-19 pandemic compared to the numbers during the previous year, and interestingly a 67% decrease in traumas, which could be explained by less outdoor and sport activities in the population [17].

At the beginning of the COVID-19 pandemic the national recommendations were to conserve resources, as a large number of health care workers were needed in the intensive care units, especially personnel from the anesthetic units. As only a limited number of anesthetists were left to staff the operation rooms, surgeries had to be prioritized to emergencies and oncology cases. There was also a shortage of personal protective equipment (PPE), making it more difficult to ensure patient and staff safety. At the same time, many hospital wards that previously treated surgical patients, shifted focus, and were used to treat patients with COVID-19 that did not need intensive care. As oral and maxillofacial surgery is performed in the oral cavity and in proximity to the nasopharynx, a higher risk of staff being infected by the highly contagious coronavirus had to be taken into consideration [18]. Taken together, these dramatic changes in the health care systems that took place in the very beginning of 2020, reflects the distinct drop in number of surgeries from March that year, as shown in this study. As more knowledge of how the virus affected populations, the healthcare of patients infected by COVID-19 improved, appropriate levels of PPE were adjusted and most importantly mass vaccinations were initiated, the numbers of OGS in Sweden were slowly raising. With time, international guidelines and recommendations started to appear in the scientific community, showing how surgery could be managed in a safe way in a pandemic situation [19]. Reports on how the COVID-19 virus affected surgical practice in oral and maxillofacial units highlighted initial difficulties with strict patient prioritization and how experiences and improved knowledge led to strategies to enable provision of care [8, 20]. Glen et al. [21] showed how working under a strict safety protocol permitted OGS during the pandemic without risking the safety of the patients or the health care personnel.

While there were significant differences between pre-pandemic and pandemic monthly OGS in the university hospitals, statistical difference could not be detected in OGS performed in the regional hospitals. The non-significant difference between the pre-pandemic and pandemic periods in regional hospitals may reflect baseline capacity and workload, and not solely pandemic-related effects. Lower sample sizes and greater variability in the data could reduce the statistical power, making it challenging to detect significant changes related to the pandemic. Additionally, in regional hospitals, an increase in surgical volumes is seen during the post-pandemic period compared to the pre-pandemic period (+ 74.9%), whereas the opposite pattern is observed in university hospitals (− 9.9%). This may indicate a redistribution of elective OGS cases toward regional hospitals following the pandemic, potentially related to surgical backlogs or capacity constraints at university hospitals. However, it is also plausible that university hospitals prioritized other elective surgical procedures during the post-pandemic period, whereas regional hospitals may have had fewer competing elective cases, thereby allowing a relative increase in OGS volumes. Furthermore, the non-significant differences between the pre- and post-pandemic periods suggests a meaningful return to pre-pandemic surgical volume levels both for regional and university hospitals. Although the difference between university and regional hospitals is most likely attributable to variations in baseline capacity and statistical power, it cannot be ruled out that the differing prioritizations and restrictions applied across Swedish regions may have affected some hospitals more than others. It is also likely that patients’ access to care was impacted, regardless of whether treatment was provided at university or regional hospitals.

During the pandemic, there was an accumulation of patients that had their surgeries postponed, which may have contributed to longer waiting times and an increased number of patients that needed surgery when the restrictions were lifted, however the effect of this phenomenon could not be observed in this study, as there were no significant differences between the pre- and the post-pandemic periods. This non-significant difference does not, however, account for surgical backlogs or waiting times, and an increased queue of elective OGS may therefore persist. Thus, although surgical volumes have returned to pre-pandemic levels, patients may still have experienced, or may continue to experience, prolonged waiting times for elective surgery.

The consequences for the patients during the pandemic period (March 2020-February 2022) were delayed operations and a prolonged treatment time in total, especially a longer time with multibracket appliance. With prolonged treatment time, the risk for complications as initial caries lesions (white spot lesions) increases if the oral hygiene is not maintained. Another consequence is a risk for emotional suffering while waiting for treatment [9, 10]. Other negative consequences of the pandemic on surgical units that needs to be taken into consideration are reduced time in the operation room for the trainees, increased anxiety, and burnout syndrome among residents [16, 22].

### Strength and limitations

This study is, to our knowledge, the first registry-based investigation to assess the impact of the COVID-19 pandemic on OGS at a national level. The data are derived from a national quality registry with a high coverage rate exceeding 90% [12], providing a reliable and comprehensive source. The findings presented in this study may be used to inform future healthcare planning, resource allocation, and strategies for maintaining elective surgical care during healthcare crises. The primary limitation of the study is its reliance on a single healthcare system, which restricts the generalizability of the findings. Another limitation is that some clinics had not yet begun to register in NROK 2018, which is the first year we collect data from. The exclusion of three hospitals may have introduced a risk of selection bias. However, these units likely represent only a small proportion of the total national volume of OGS. Two of the excluded hospitals were affiliated with NROK but had no registered cases during the study period, most likely due to reporting negligence rather than a complete absence of surgical activity. Nevertheless, the surgical volumes at these units were presumably too low to substantially affect the overall findings. Reoperations were included in the final analysis of monthly mean OGS volumes; however, the relatively low proportion (4.5%) is unlikely to have materially affected the results and should be considered within the scope of total surgeries performed. As seen in Fig. 4, one hospital (University Hospital 5) accounted for nearly one third of all OGS performed during the pandemic period and demonstrated a volume pattern distinct from other university hospitals. Although exclusion of this outlier would have strengthened the observed pandemic effect, exclusion was not justified in a national registry–based study. This should be considered when interpreting the aggregated results.

In a study on OGS in Sweden performed before the NROK registry was launched, data extracted from the Swedish National Board of Health and Welfare register showed a variation between regions in the country, with a higher number of OGS performed in the Southern region [3]. Comparisons between regions have not been made in this study, only between regional and university hospitals, although the effect of the pandemic could have varied throughout the country depending on local political decisions or available medical resources. Comparisons of results worldwide are associated with some difficulties due to the differences in healthcare systems. In Sweden, all OGS are performed in public hospitals which facilitates assessments of how surgical treatments of dentofacial deformities are affected by events in society and the implimentation of the NROK registry has greatly improved these possibilities. Lastly, another potential limitation is the age restriction, as only individuals aged 18 years or older were included. This was due to ethical approval constraints, which did not justify the inclusion of minors. However, in Sweden, candidates for OGS are rarely operated on before the age of 18. Therefore, the risk of introducing selection bias should be negligible.

### How can we use these results?

The cancellation and postponement of elective surgeries in Sweden during to the COVID-19 pandemic had negative consequences such as prolonged treatment times for patients, emotional distress and reduced training opportunities for staff. These findings highlight the need for improved strategies for resource allocation during crises. Specifically, frameworks for redistributing recourses could be developed to ensure continuity of elective surgeries while addressing critical care demands.

One potential implication, although it must be interpreted with caution, is that, during national crises, some elective surgical activity might be redistributed to regional hospitals. In this study, regional hospitals showed a smaller proportional reduction in OGS volume; however, we cannot determine whether this reflects differences in pandemic impact, baseline capacity, or other contextual factors. It should be emphasized that this interpretation is context-specific to the Swedish healthcare system, where university hospitals manage both highly specialized care and regional inpatient responsibilities. As this organizational structure differs internationally, the generalizability of this reasoning is limited. Nevertheless, proactive planning and clear national guidelines will be important to mitigate the impact of future crises on elective surgery.

Future studies should specifically examine whether earlier or ongoing COVID-19 infection influences postoperative outcomes in orthognathic surgery—such as complication rates, reoperation rates, hospital length of stay, or the optimal timing of surgery following recovery—as these clinically important factors could not be assessed with the present dataset.

## Conclusions

- A significant decrease in the number of orthognathic surgeries was observed in Sweden during the COVID-19 pandemic.
- Comparisons of pre- and post-pandemic monthly mean surgery volumes showed no statistically significant differences, suggesting a meaningful return to pre-pandemic levels of OGS activity in Sweden. This does not account for potential waiting times for elective OGS that may still persist following the pandemic.
- University hospitals experienced a larger proportional decline in surgical volume during the pandemic and a slower recovery in the post-pandemic period compared with regional hospitals.
- While these findings highlight differences in how hospital types were affected, any implications for future resource allocation should be interpreted with caution and in light of the study’s limitations. Nevertheless, the results may contribute to preparedness planning for maintaining elective surgical care during future healthcare disruptions.

## Data Availability

The registry data delivered by NROK is restricted to use only by the researchers included in the ethical approval and can therefore not be available to third party.

## Abbreviations

NROK: National Registry for Orthognathic Surgery
OGS: Orthognathic Surgery
PPE: Personal Protective Equipment
PHAS: Public Health Agency of Sweden
SARME: Surgically Assisted Rapid Maxillary Expansion
IVRO: Intraoral Vertical Ramus Osteotomies
EVRO: Extraoral Vertical Ramus Osteotomies
BSSO: Bilateral Sagittal Split Osteotomy
COVID-19: Coronavirus Disease 2019
